# Determinants of Survival After Sorafenib Failure in Patients With BCLC-C Hepatocellular Carcinoma in Real-World Practice

**DOI:** 10.1097/MD.0000000000000688

**Published:** 2015-04-10

**Authors:** I-Cheng Lee, Yi-Tzen Chen, Yee Chao, Teh-Ia Huo, Chung-Pin Li, Chien-Wei Su, Han-Chieh Lin, Fa-Yauh Lee, Yi-Hsiang Huang

**Affiliations:** From the Division of Gastroenterology (I-CL, T-IH, C-PL, C-WS, H-CL, F-YL, Y-HH), Department of Medicine, Taipei Veterans General Hospital; Institute of Clinical Medicine (I-CL, Y-HH), National Yang-Ming University School of Medicine; Department of Nursing (Y-TC), Taipei Veterans General Hospital; Cancer Center (YC), Taipei Veterans General Hospital; and Institute of Pharmacology (T-IH), National Yang-Ming University School of Medicine, Taipei, Taiwan.

## Abstract

Sorafenib may improve progression-free survival (PFS) and overall survival (OS) of advanced hepatocellular carcinoma (HCC). However, the survival benefit is short lived and survivals after progressive disease (PD) have not been well characterized. This study aimed to evaluate the survival predictors of OS and postprogression survival (PPS) in advanced HCC patients receiving sorafenib treatment.

Consecutive 149 HCC patients receiving sorafenib under National Health Insurance were retrospectively enrolled. All patients fulfilled the reimbursement criteria: Barcelona Clinic Liver Cancer stage C HCC with macroscopic vascular invasion or extrahepatic metastasis (Mets), and Child–Pugh class A. Radiologic assessment was performed at a 2-month interval using modified Response Evaluation Criteria in Solid Tumors. Patients who maintained Eastern Cooperative Oncology Group ≤2 and Child–Pugh class A at PD were assumed to be candidates for second-line treatment.

During the median follow-up period of 7.5 months (range, 1.1–18.5), PD developed in 120 (80.5%) patients and 96 (64.4%) deaths occurred. The median PFS, OS, and PPS were 2.5, 8.0, and 4.6 months, respectively. In general, patients with Mets only had better OS and PPS than those with portal vein invasion. Independent predictors of OS include baseline performance status (hazard ratio [HR] = 1.956), tumor size (HR = 1.597), alpha-fetoprotein (HR = 1.869), discontinuation of sorafenib due to liver function deterioration (LD) (HR = 6.142), or concurrent PD and LD (HR = 2.661) and PD within 4 months (HR = 5.164). Independent predictors of PPS include deteriorated performance status (HR = 7.680), deteriorated liver functions (HR = 5.603), bilirubin (HR = 2.114), early PD (HR = 6.109), and new extrahepatic lesion (HR = 1.804). In 46 candidates for second-line trials, development of new extrahepatic lesion independently predicts poorer PPS (HR = 3.669).

In conclusion performance status, liver functions, early disease progression, and progression pattern are important determinants of survival after sorafenib failure. These factors should be considered in clinical practice and second-line trial designs for patients with sorafenib failure.

## Abstract 2

Supplemental Digital Content is available in the text

## INTRODUCTION

Hepatocellular carcinoma (HCC) is the sixth most common cancer and third leading causes of cancer-related death worldwide.^1,2^ Its prevalence is high in hepatitis B virus (HBV) and hepatitis C virus (HCV) endemic areas, and its incidence is still rising.^2^ Despite advances in many aspects of HCC surveillance and treatment, the majority of patients still present with an already advanced stage, in whom the prognosis is poor and treatment options are limited.^3–7^

Sorafenib, a multikinase inhibitor that targets multiple signaling pathways and inhibits tumor-cell proliferation and tumor angiogenesis, has been shown to prolong both progression-free survival (PFS) and overall survival (OS) in advanced HCC.^8,9^ Although the disease control with sorafenib is short lived and the median survival rate is still <1 year, it is still the only approved systemic agent for advanced HCC. For patients who developed progressive disease (PD) after sorafenib treatment, sorafenib was often discontinued and second-line trials would be considered.^10^ Currently, the impact of progression pattern on survival after sorafenib treatment failure has not been well characterized, especially for those who are candidates for second-line treatment, and identifying survival predictors of postprogression survival (PPS) may have impact on the clinical management as well as second-line trials design. A recent study by Reig et al^11^ demonstrated the PPS in HCC patients receiving sorafenib treatment and showed that the progression pattern influenced both OS and PPS. However, more than half of the studied cases were Barcelona Clinic Liver Cancer (BCLC) stage B. Moreover, the efficacy of sorafenib for HCC in Asian-Pacific region differed from Western countries,^8,9^ and the predictors of survival after PD would vary in the East.

In Taiwan, under the regulations of the National Health Insurance Administration, Ministry of Health and Welfare, sorafenib was reimbursed for patients with BCLC-C HCC since August 2012. Patients receiving sorafenib treatment under the National Health Insurance program should have close and regular monitoring when applying further course of sorafenib reimbursement, and thus this cohort of patients could be well evaluated for the treatment efficacy of sorafenib in the real-world setting. In this study, we aimed to evaluate the survival predictors of PFS, OS, and PPS in BCLC-C HCC patients receiving sorafenib treatment. The potential influence of progression pattern on survivals was also analyzed.

## MATERIAL AND METHODS

### Patients

From August 2012 to May 2013, consecutive advanced HCC patients in Taipei Veterans General Hospital, Taipei, Taiwan, who received sorafenib treatment under the reimbursement of National Health Insurance program were retrospectively enrolled. The reimbursement criteria included BCLC stage-C HCC accompanied with macroscopic major vascular invasion (VI) focusing on Vp3 or Vp4 or extrahepatic metastasis (Mets); an Eastern Cooperative Oncology Group (ECOG) performance status score of ≤2; and Child–Pugh liver function class A. Patients should fulfill all the 3 criteria and the clinical data were reviewed by the National Health Insurance Administration prior to the approval of sorafenib reimbursement. Patients who had received previous local therapy, such as surgery, radiofrequency ablation, percutaneous injection, transarterial chemoembolization (TACE), transarterial radioembolization (TARE), or radiotherapy were eligible for enrolment in the reimbursement program. At least 1 untreated target lesion that could be measured in 1 dimension was selected to evaluate the response according to the modified Response Evaluation Criteria in Solid Tumors (mRECIST).^12,13^ Concomitant antiviral systemic therapy was allowed. This study was approved by the Institutional Review Board, Taipei Veterans General Hospital, which complied with standards of the Declaration of Helsinki and current ethical guidelines.

The diagnosis of HCC was made mainly based on imaging modalities using contrast-enhanced computed tomography (CT), magnetic resonance image (MRI), and/or pathologically by tumor biopsy, which fulfilled the diagnostic criteria of the American Association for the Study of Liver Diseases (AASLD) treatment guidelines for HCC.^4^ Radiologic tumor progression was confirmed by contrast-enhanced CT or MRI.

All patients received 400 mg of sorafenib (consisting of two 200-mg tablets) twice daily. Sorafenib dosage was modified upon development of adverse events according to the manufacturer's recommendations. Treatment continued until the occurrence of radiologic progression, as defined by mRECIST, or the occurrence of deteriorated liver functions to Child–Pugh class B or C, or the occurrence of either unacceptable adverse events or death.

### Assessments

Under the regulations of the National Health Insurance Administration, tumor measurements were performed at screening and every 2 months during treatment by contrast-enhanced CT or MRI. Patients visited the clinic every 2 to 4 weeks for assessment of compliance and determination of side effects. Compliance was assessed on the basis of pill counts and diary entries of patients. If PD or deteriorated liver functions to Child–Pugh class B or C were noted, further reimbursement of sorafenib was not allowed. Tumor status, ECOG status, Child–Pugh score, serum alpha-fetoprotein (AFP) level, and serum biochemistry were reevaluated at the time of radiologic progression. Patients who maintained their performance status (ECOG ≤ 2) and liver functions (Child–Pugh class A) at the time of PD were assumed to be candidates for second-line treatment.^14^

The survival status of the study patients was obtained from hospital records. Local response was determined by the mRECIST criteria: complete response (CR)—disappearance of any intratumoral arterial enhancement in all target lesions; partial response (PR)—at least a 30% decrease in the sum of the diameters of viable target lesions; PD—an increase of at least 20% in the sum of the diameters of viable target lesions; and stable disease (SD)—any cases that do not qualify for either PR or PD.^12^

### Outcomes

PFS was defined as the time from starting sorafenib treatment to disease progression. Patients who died before the first imaging assessment were classified as progressors.^11^ OS was defined as the time from starting sorafenib treatment to death from any cause.^15^ PPS was defined as the time from disease progression to death. The disease-control rate was defined as the percentage of patients who had a best-response rating of CR, PR, or SD at 2 and 4 months after sorafenib treatment. The patterns of tumor progression were classified into intrahepatic or extrahepatic tumor growth (>20% increase in tumor size of the viable target lesions), new intrahepatic lesions, and new extrahepatic lesions (including new metastasis and/or VI).^11^

### Clinical and Laboratory Data

The following variables were used for analysis: age, sex, ECOG performance status, intrahepatic tumor size, macroscopic VI (Vp3 or Vp4), Mets, serum levels of AFP, alanine aminotransferase (ALT), aspartate aminotransferase (AST), albumin, total bilirubin and creatinine, prothrombin time (measured by international normalized ratio [INR]), hepatitis B surface antigen (HBsAg,) and anti-HCV. Serum HBsAg and serum AFP were measured by radioimmunoassay kits (Abbott Laboratories, North Chicago, IL, and Serono Diagnostic SA, Coinsin/VD, Switzerland, respectively). Anti-HCV was detected by an enzyme immunoassay kit (Abbott Laboratories), whereas serum albumin, ALT, AST, total bilirubin, and creatinine were measured by systemic multiautoanalyzer (Technicon SMAC, Technicon Instruments Corp., Tarrytown, NY).

### Statistical Analysis

Values were expressed as median (ranges) or as mean ± standard deviation when appropriate. The Pearson χ^2^ analysis or Fisher exact test was used to compare categorical variables, whereas the Student *t* test or Mann–Whitney *U* test was used for continuous variables. Survival was estimated by the Kaplan–Meier method and compared by the log-rank test. Analysis of prognostic factors for PFS, OS, or PPS was performed using the Cox proportional hazards model. Variables that achieved statistical significance (*P* < 0.05) or those that were close to significance (*P* < 0.1) by univariate analysis were subsequently included in the forward stepwise multivariate analysis. A 2-tailed *P* < 0.05 was considered statistically significant. The cutoff values for clinical variables were chosen according to clinically significant values. All statistical analyses were performed using the Statistical Package for Social Sciences (SPSS 17.0 for Windows, SPSS Inc., Chicago, IL).

## RESULTS

### Baseline Characteristics

From August 2012 to May 2013, consecutive 149 patients with BCLC stage-C HCC receiving sorabenib treatment by National Health Insurance program were enrolled. Baseline characteristics of the 149 patients were summarized in Table 1. Chronic HBV infection was the predominant cause of liver disease, followed by chronic HCV infection and alcohol consumption. Majority of the patients (78.5%) were rated as ECOG score 0 at baseline, reflecting well-preserved performance status. At baseline, 99 patients (66.4%) had macroscopic VI and 92 (61.7%) had Mets, with the most common extrahepatic sites being lung, lymph node, bone, and adrenal gland. Forty-two patients (28.2%) had concurrent VI and Mets. Fifty-nine of the 85 patients (69.4%) with chronic HBV infection were under nucleos(t)ide analogues antiviral therapy before starting sorafenib treatment.

**TABLE 1:**
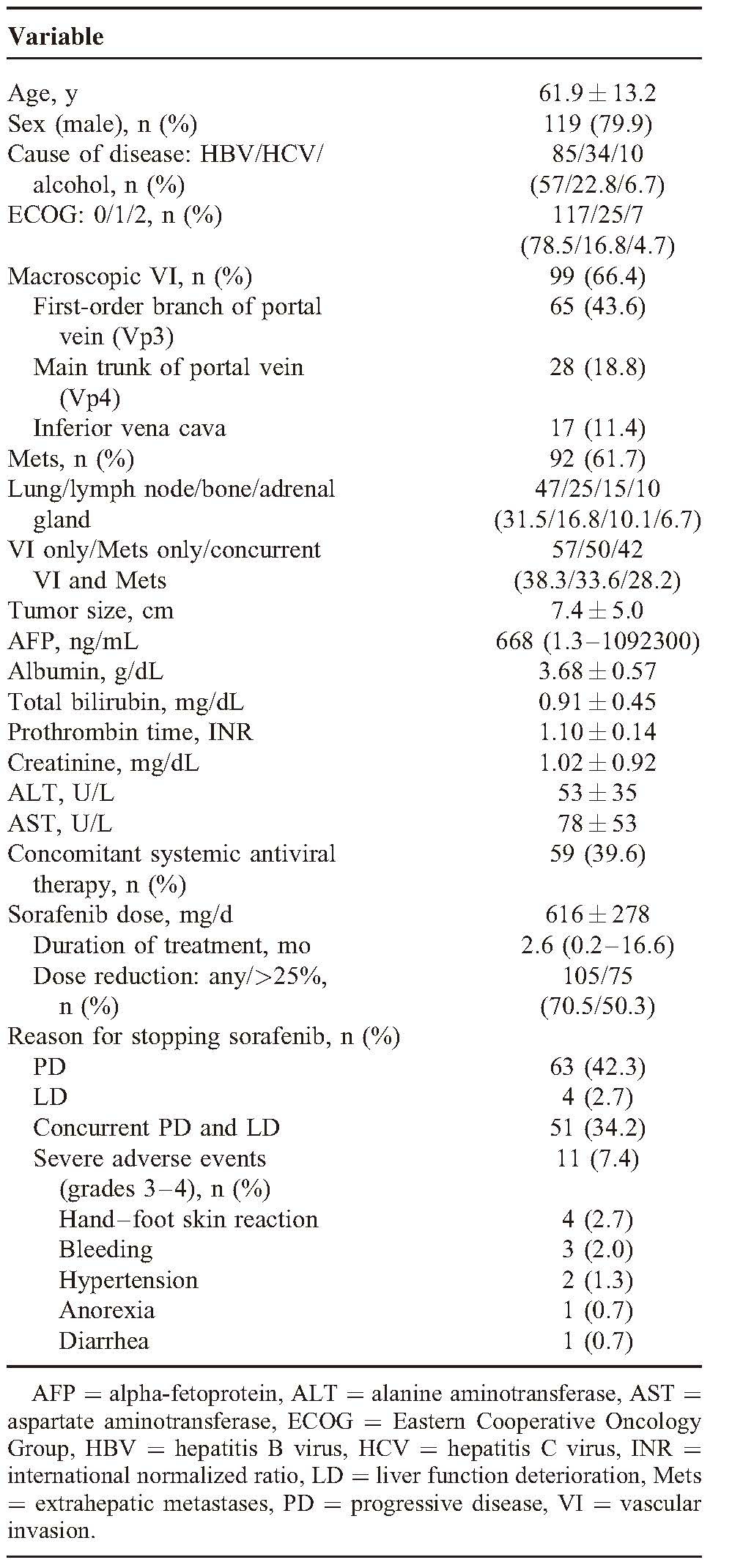
Baseline Characteristics of the 149 Patients Receiving Sorafenib Treatment

### Treatment, Tolerability, and Adverse Events

The median duration of sorafenib treatment was 2.6 months (range, 0.2–16.6) and the mean dose of sorafenib was 616 mg/d (Table 1). Overall, 105 patients (70.5%) experienced dose reduction and 75 patients (50.3%) received <75% of the planned daily dose during the treatment. The main reasons for discontinuation of sorafenib were PD (42.3%), concurrent PD and liver function deterioration (LD) (34.2%), and severe adverse events (7.4%). Hand–foot skin reaction was the most common severe adverse events leading to discontinuation.

### Overall Outcomes

**FIGURE 1:**
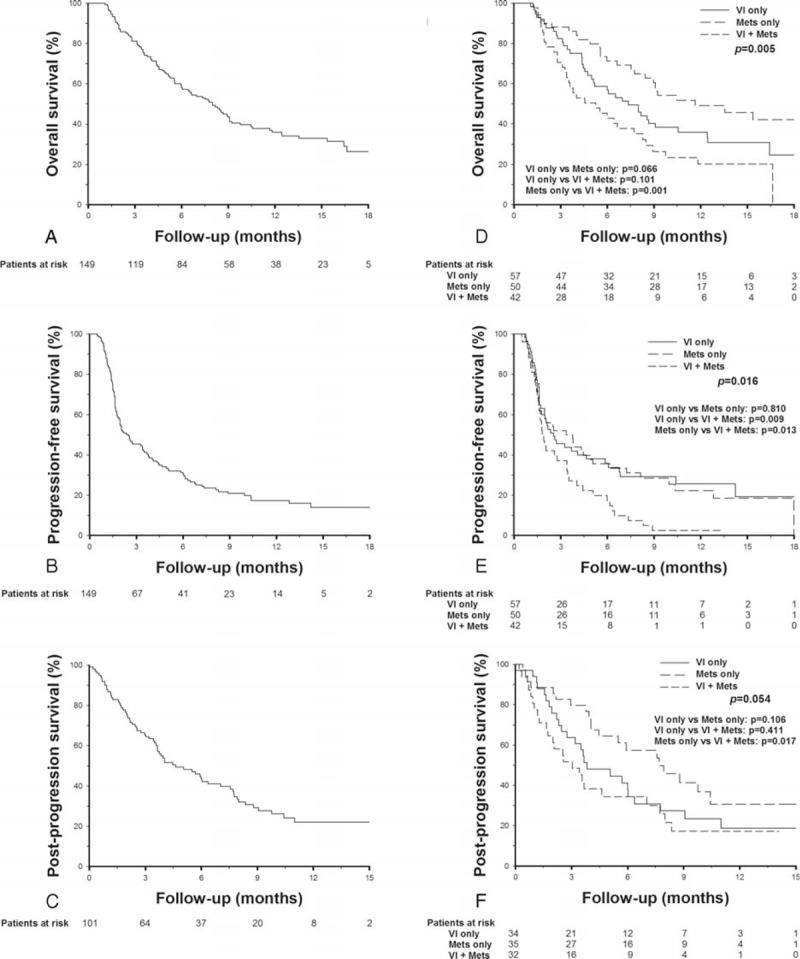
Kaplan–Meier survival curves in HCC patients receiving sorafenib treatment. (A) Overall survival. (B) Progression-free survival (C) Postprogression survival. (D) Overall survival stratified by initial tumor status. (E) Progression-free survival stratified by initial tumor status. (F) Postprogression survival stratified by initial tumor status. HCC = hepatocellular carcinoma.

The treatment outcomes were summarized in Table 2. During the median follow-up period of 7.5 months (range, 1.1–18.5), 96 (64.4%) deaths occurred and the median OS was 8 months (Figure 1A). The estimated 6-month and 1-year OS rates were 59.3% and 36.1%, respectively. Liver failure was the leading cause of death (53.1%), followed by tumor progression (34.4%) and infection (9.4%).

**TABLE 2:**
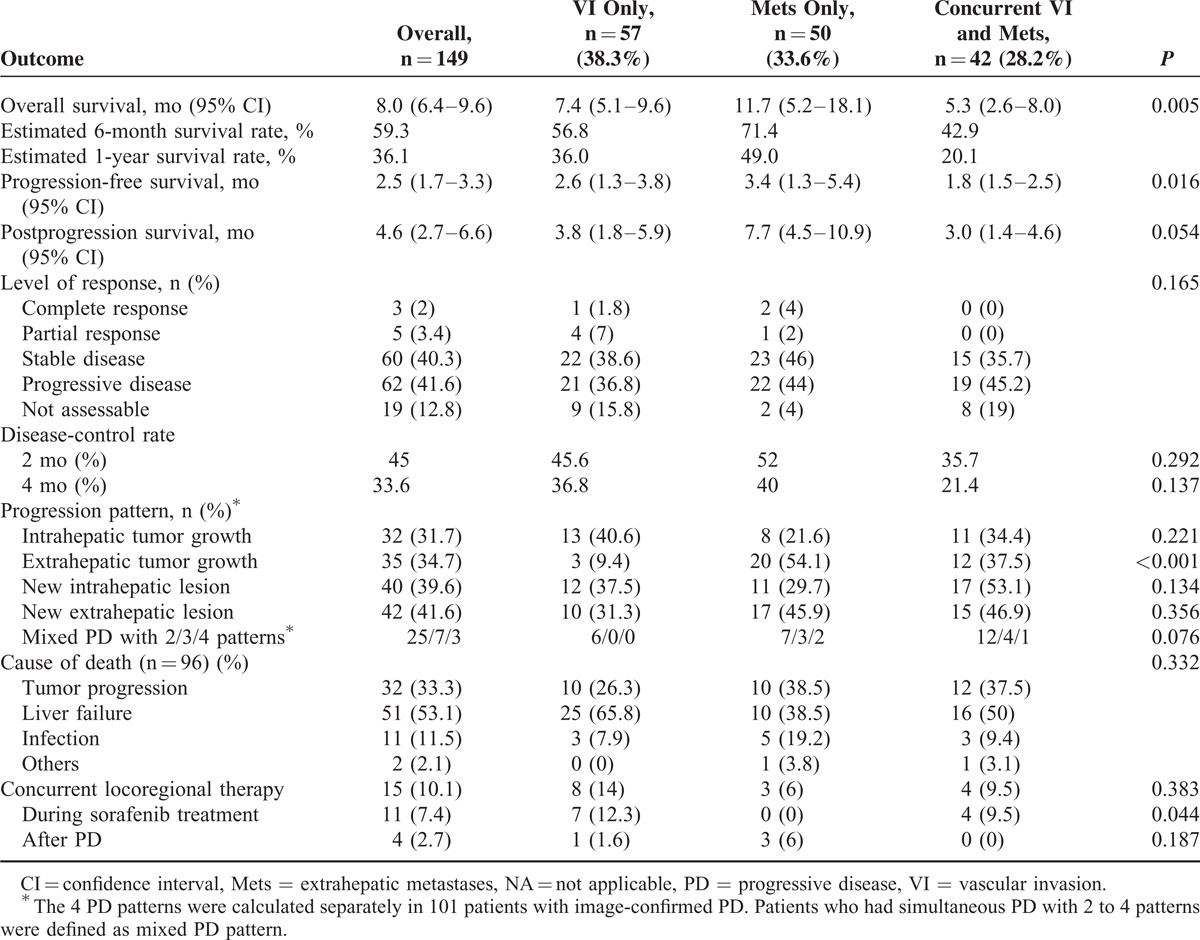
Summary of Treatment Outcomes According to Tumor Status

PD occurred in 120 (80.5%) patients, including 19 deaths before the first-image reevaluation and 101 patients with image-confirmed PD. The median PFS was 2.5 months (Figure 1B). In patients with image-confirmed PD, 32 (31.7%) had intrahepatic tumor growth, 35 (34.7%) had extrahepatic tumor growth, 40 (39.6 %) had new intrahepatic lesions, and 42 (41.6 %) had new extrahepatic lesions. Thirty-five patients (34.7%) had mixed presentation of either 4 types of tumor progression. The median PPS of the 101 patients with image-confirmed PD was 4.6 months (Figure 1C). Fifteen patients (10.1%) received concurrent locoregional therapy or rescue therapy during the follow-up period, including 11 cases with concurrent locoregional therapy (TACE, n = 6; TARE, n = 5) during sorafenib treatment and 4 cases with rescue therapy after PD (TACE, n = 3; chemotherapy, n = 1).

In the analysis for best response, 3 patients (2%) had CR, 5 (3.4%) had PR, and 59 (39.6%) had SD. The 2 and 4-month disease-control rates were 45% and 33.6%, respectively (Table 2).

### Outcomes According to Initial Tumor Status

The median OS was 7.4, 11.4, and 5.3 months (*P* = 0.005), respectively, in patients with VI only, Mets only, and concurrent VI and Mets (Table 2). Patients with Mets only had a trend of better OS as compared to patients with VI only (*P* = 0.066), and had a significantly better OS as compared to patients with concurrent VI and Mets (*P* = 0.001, Figure 1D). The median PFS was 2.6, 3.4, and 1.8 months (*P* = 0.016), respectively, in patients with VI only, Mets only, and concurrent VI and Mets, respectively. The PFS were comparable between patients with VI only and Mets only (*P* = 0.810), and were significantly poorer in patients with concurrent VI and Mets (*P* < 0.05, Figure 1E). The median PPS was 3.8, 7.7, and 3 months (*P* = 0.054), respectively, in patients with VI only, Mets only, and concurrent VI and Mets. Patients with Mets only at initial presentation also had a better PPS as compared to patients with concurrent VI and Mets (*P* = 0.017, Figure 1F). The level of response were not significantly different among the 3 groups of patients based on their initial tumor status, but patients with concurrent VI and Mets had a slightly lower 2 and 4-month disease-control rate after sorafenib treatment (Table 2).

### Factors Associated With PFS

Concurrent VI and Mets, tumor size, serum AST levels, and sorafenib dose reduction were factors associated with PFS by univariate analysis. In multivariate analysis, sorafenib dose reduction (hazard ratio [HR] = 0.421, *P* < 0.001) and elevated AST levels (HR = 1.786, *P* = 0.006) were independent predictors of PFS (Table 3).

**TABLE 3:**
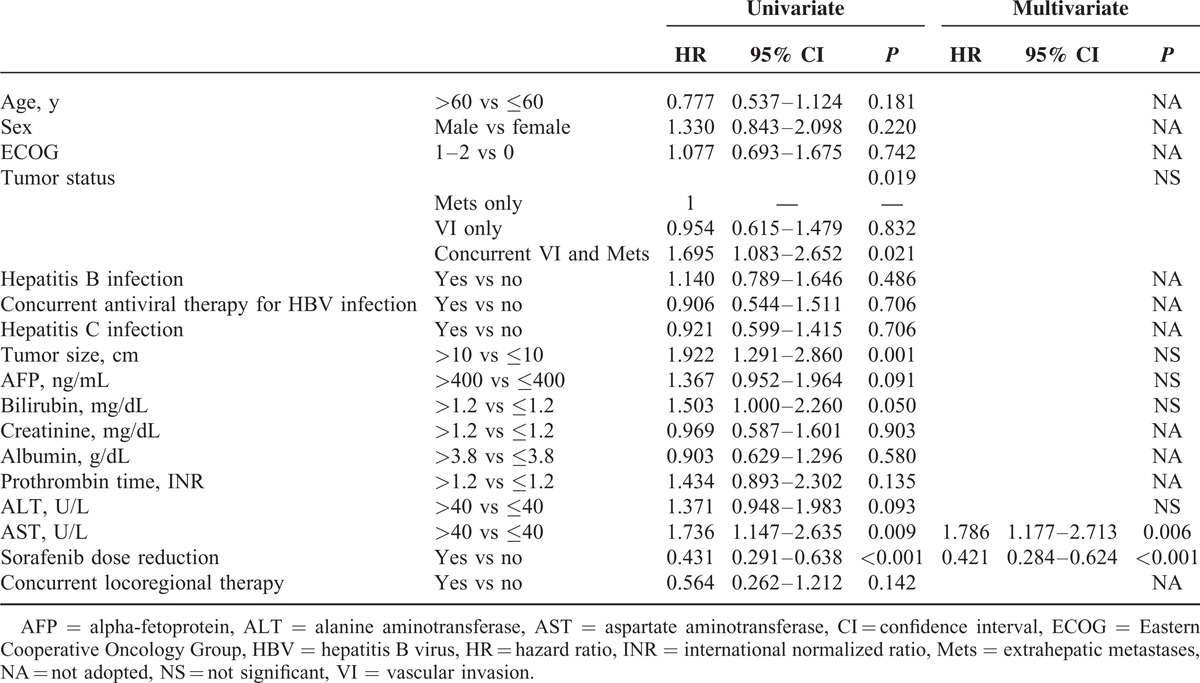
Univariate and Multivariate Analyses of Factors Associated With Progression-Free Survival

### Factors Associated With OS

**FIGURE 2:**
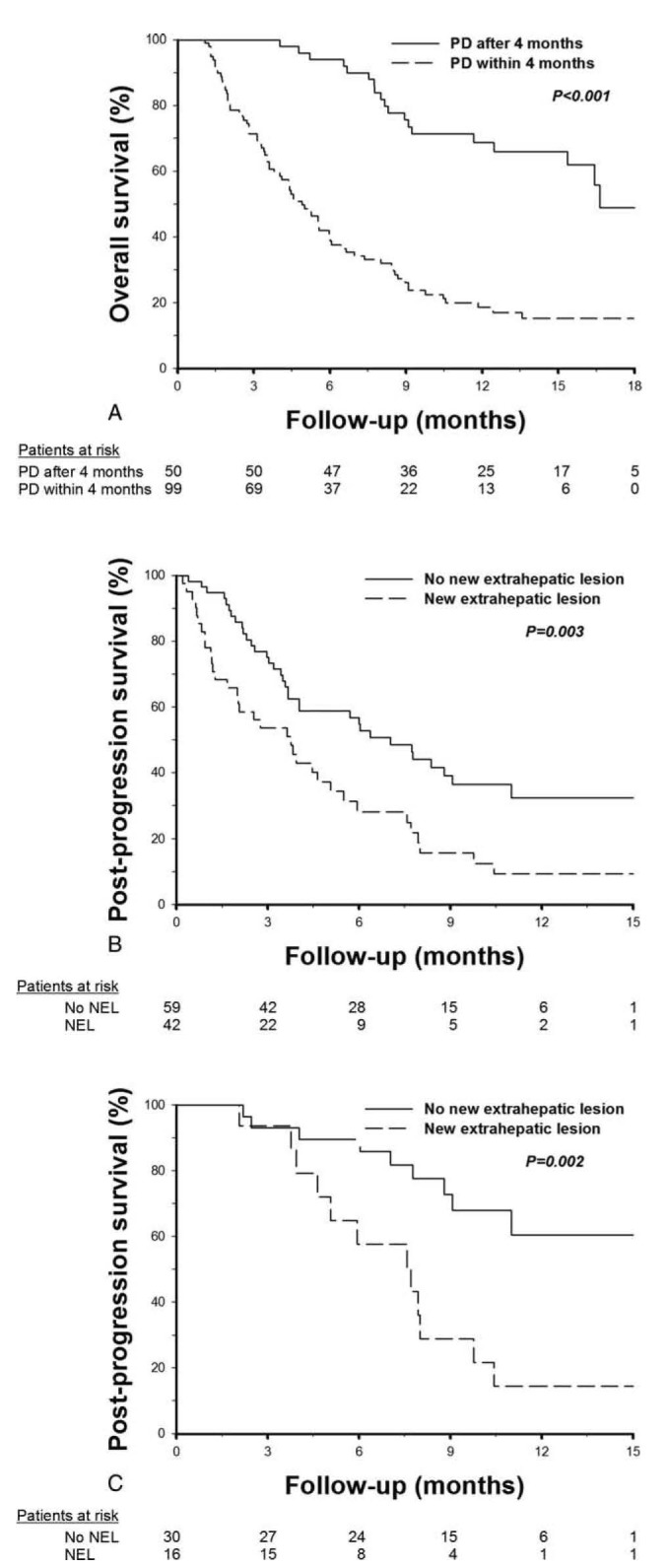
Kaplan–Meier analysis of overall and postprogression survivals in patients with different progression pattern. (A) Overall survival in patients with and without early disease progression. (B) Postprogression survival in 101 patients with image-confirmed disease progression. (C) Postprogression survival in 46 candidates of second-line treatment. NEL = new extrahepatic lesion.

In univariate analysis, predictors of OS include ECOG performance status, concurrent VI and Mets, tumor size, serum AFP, bilirubin, albumin, AST levels, reasons for discontinuation of sorafenib, and PD within 4 months (Table 4). The median OS in patients who discontinued sorafenib due to PD only, LD only, concurrent PD and LD, and severe adverse events were 9.1, 5.6, 4.4, and 6.7 months, respectively (*P* < 0.001). Patients with early PD had significantly worse OS than those with PD after 4 months (median OS 4.9 vs 16.6 months, *P* < 0.001, Figure 2A). In multivariate analysis, ECOG performance statuses 1–2 (HR = 1.956, *P* = 0.004), tumor size >10 cm (HR = 1.597, *P* = 0.049), AFP >400 ng/mL (HR = 1.869, *P* = 0.008), discontinuation of sorafenib due to LD (HR = 6.142, *P* = 0.002) or concurrent PD and LD (HR = 2.661, *P* < 0.001), and PD within 4 months (HR = 5.164, *P* < 0.001) were independent factors associated with OS.

**TABLE 4:**
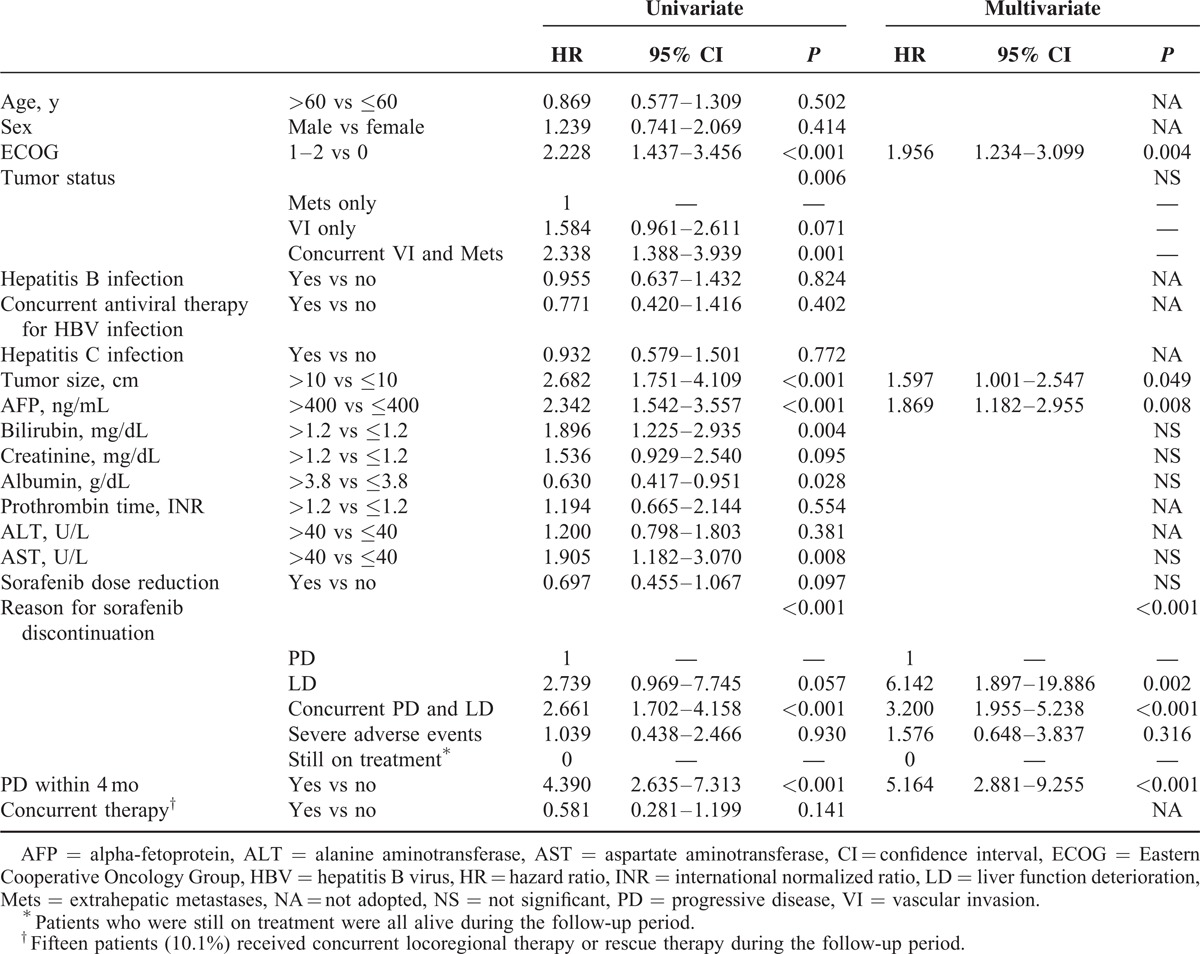
Univariate and Multivariate Analyses of Factors Associated With Overall Survival

### Factors Associated With PPS

Patient conditions were reevaluated at the time of PD for survival analysis of PPS. In the 101 patients with image-confirmed PD, 27 (26.7%) patients had deteriorated performance status to ECOG 3–4, whereas 36 (35.6%) and 15 (14.9%) had deteriorated liver functions to Child–Pugh classes B and C, respectively. In univariate analysis, reasons for discontinuation of sorafenib, ECOG, Child–Pugh class, concurrent VI and Mets, tumor size, serum bilirubin, creatinine, albumin, prothrombin time, and AST levels at the time of PD, PD within 4 months, development of intrahepatic growth, and new extrahepatic lesion were factors associated with PPS (Table 5). The median PPS in patients who discontinued sorafenib due to PD only (n = 50), concurrent PD and LD (n = 47), and severe adverse events before PD (n = 4) were 8.8, 2.3, and 3.7 months, respectively (PD only vs concurrent PD and LD: *P* < 0.001). In multivariate analysis, ECOG 3–4 (HR = 7.680, *P* < 0.001), Child–Pugh class B or C (HR = 5.603, *P* < 0.001), bilirubin >1.2 mg/dL (HR = 2.114, *P* = 0.012), PD within 4 months (HR = 6.109, *P* < 0.001), and development of new extrahepatic lesion (HR = 1.804, *P* = 0.021) were independent predictors of PPS (Table 5). The median PPS in patients with and without new extrahepatic lesion was 3.7 and 7 months, respectively (*P* = 0.003, Figure 2B).

**TABLE 5:**
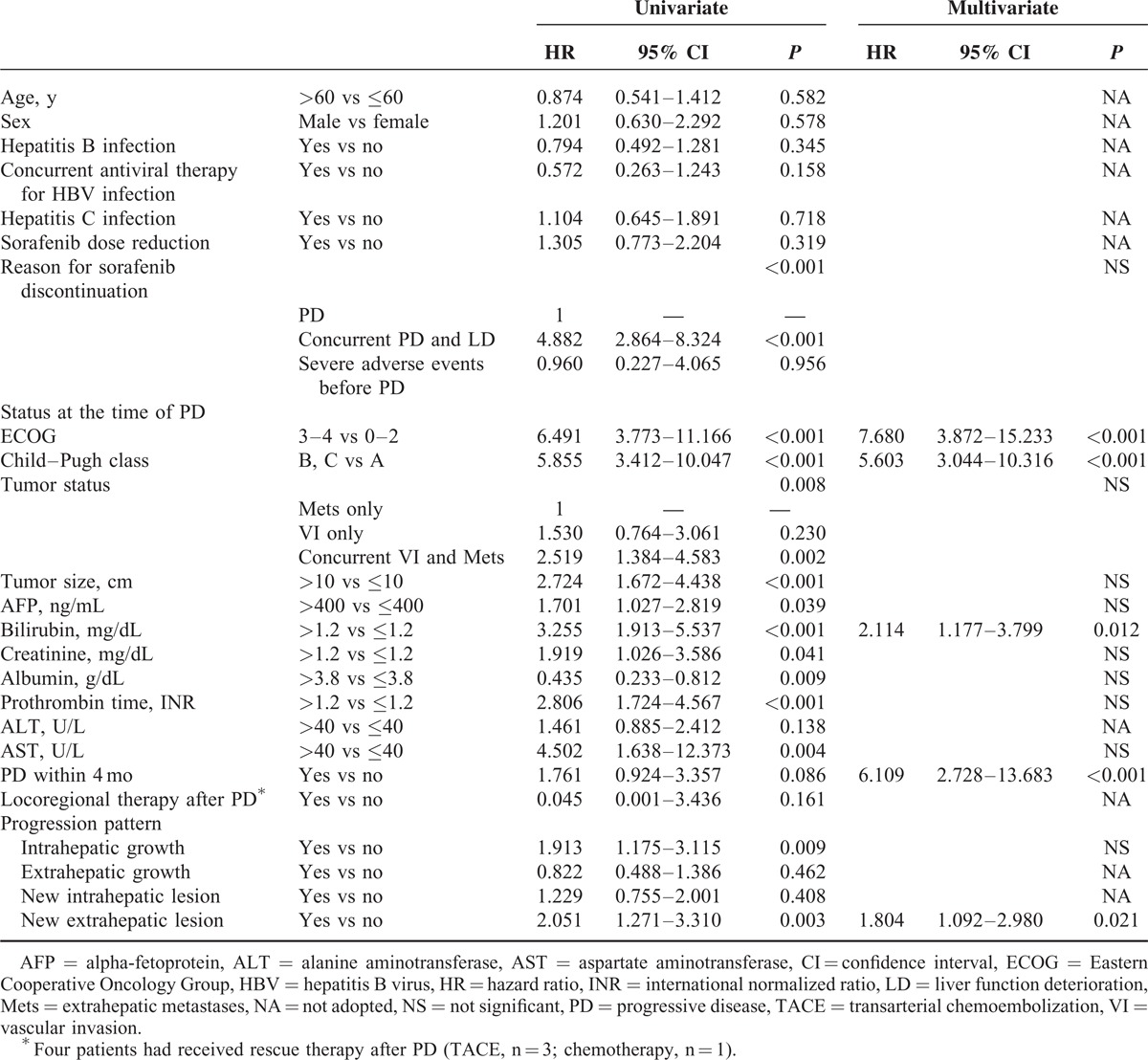
Univariate and Multivariate Analyses of Factors Associated With Postprogression Survival in 101 Patients With Image-Confirmed PD

### Factors Associated With PPS in Patients Who Assumed to Be Candidates for Second-Line Treatment After PD

At the time of PD, 46 (45.5%) patients maintained well performance status (ECOG ≤ 2) and liver functions (Child–Pugh class A) and were considered to be candidates for second-line treatment after PD. The median PPS in these patients was 10.4 months. In univariate analysis, bilirubin and new extrahepatic lesion were predictors of PPS (Supplementary Table 1, http://links.lww.com/MD/A242). In multivariate analysis, new extrahepatic lesion was the only independent predictor of PPS (HR = 3.669, *P* = 0.003, Figure 2C).

## DISCUSSION

Under the strict reimbursement criteria of National Health Insurance program in Taiwan, only BCLC stage-C HCC patients with Child–Pugh class A at baseline were enrolled in this study. Therefore, our patient population had more homogeneous tumor stage and liver condition than those in the previous phase III trials, in which some patients with BCLC stage B and Child–Pugh class B were also enrolled. Our results showed that the efficacy of sorafenib for HCC in the real-world setting, including PFS, OS, and response rates, was comparable to that reported in the previous phase 3 trials (Table 2), which supports the current policy to reimburse sorafenib by National Health Insurance program.

The median PFS of 2.5 months in this study was similar to the results of the Asian-Pacific trial, reflecting the poorer treatment response in the East (Table 2). In univariate analysis, concurrent VI and Mets, larger tumor size, and higher AST levels were baseline predictors of PPS, suggesting that larger tumor burden at baseline was hard to be controlled by sorafenib monotherapy. On the other hand, on-treatment sorafenib dose reduction independently predicts a better PFS, which was consistent with previous reports that dose reduction during sorafenib treatment correlated with a better survival.^11,16,17^

The median OS was 8 months, and only about one third of patients survived over 1 year. ECOG performance status, concurrent VI and Mets, tumor size, serum AFP, bilirubin, creatinine, albumin, and AST levels were baseline factors associated with OS. Performance status has been shown to correlate strongly with both tumor and cirrhotic factors and may predict survival outcome in advanced HCC patients.^18,19^ The finding that patients with Mets only had longer OS might be due to the lower rates of mortality by liver failure in patients with Mets than those with VI only, whereas in patient with concurrent VI and Mets, the larger tumor burden and more extensive tumor spread may explain the poorer outcome in these patients. Tumor size and AFP, components of Cancer of the Liver Italian Program staging system, also have been shown to correlate with OS in HCC patients receiving sorafenib treatment.^20–22^ Similarly, bilirubin and creatinine were components of the MELD score and could predict the prognosis of chronic liver disease.^23^ Previous studies also identified AST as a predictive marker in HCC patients receiving sorafenib treatment.^19,24^ Consistent with previous report that early radiologic progression after sorafenib treatment predicts poorer survival, our patients who developed early PD also had significantly worse OS.^16^

PPS in HCC patients after sorafenib treatment failure was less well characterized in Asians. The median PPS of 4.6 months in this study was similar to another recent study from Taiwan recruiting patients from clinical trials,^25^ but poorer than the report by Reig et al^11^ from Western countries. At the time of PD, significant proportion of patients had deterioration of performance status and liver function, and these 2 factors were also independent predictors of PPS. Other factors associated with PPS in univariate analyses were similar to the survival predictors of OS, including concurrent VI and Mets, tumor size, AFP, bilirubin, creatinine, albumin, prothrombin time, and AST, indicating that tumor factors and liver and renal functions all had impact on both OS and PPS. Early PD also predicts poorer PPS after adjusting for other survival predictors. In other words, patients who developed PD earlier also died earlier after PD. The median PPS of patients who failed to achieve initial disease control was 3.9 months and these patients should be referred for second-line treatment earlier.

A recent study by Iavarone et al^26^ showed that the reasons for discontinuation of sorafenib treatment correlated with different outcome in patients with advanced HCC. In the study, 30%, 47%, and 23% of patients discontinued sorafenib treatment due to adverse events, tumor progression, and liver decompensation, and the median post-sorafenib survival of the 3 groups were 4.6, 7.3, and 1.8 months, respectively.^26^ Consistent with the previous report, we also demonstrated that patients who discontinued sorafenib due to concurrent PD and liver failure had significantly worse OS and PPS than those with PD only. Although the reason for discontinuation was not an independent predictor of PPS by multivariate analysis, if we excluded Child–Pugh liver function class and total bilirubin level at the time of PD in the multivariate analysis, then the reasons for discontinuation would be an independent predictor of PPS. The findings support that liver reserve is an important survival factor for advanced HCC patients.

It is worthy to note that the development of intrahepatic tumor growth and new extrahepatic lesion correlated with poorer PPS, and new extrahepatic lesion remains independent predictor of PPS by multivariate analysis. Compared to patient with PD other than intrahepatic growth, our patient with intrahpetic tumor progression had significantly larger size of primary lesion (8.8 vs 5.9 cm, *P* = 0.001). As the intrahepatic tumor size enlarged after PD, the tumor burden further increased could lead to a worse prognosis. The finding that new extrahepatic lesion correlated with PPS was consistent with the report by Reig et al.^11^ In these patients with new metastasis and/or VI, the tumor aggressiveness failed to be controlled by sorafenib, and the widespread tumor spreading might result in the dismal outcome. The prominent role of PD pattern in PPS suggests that these radiologic progression patterns should be taken into consideration for predicting patient outcomes in clinical practice. Although few patients received concurrent locoregional therapy or rescue therapy during the follow-up period, no significant survival benefits regarding PFS, OS, and PPS were observed.

After PD, about half of patients had deteriorated performance status (ECOG > 2) and liver functions (Child–Pugh score > 6), and the outcome of them was significantly worse than those who maintained the general condition well (median PPS 2.5 vs 10.4 months, *P* < 0.001). In the 46 potential candidates for second-line treatment, predictors of PPS include bilirubin and the development of new extrahepatic lesion. Tumor progression due to new extrahepatic lesion still determined PPS in this subgroup of patients. Our cohort of 149 patients was more than twice in number of BCLC-C HCC cases of the study by Reig et al^11^ with similar findings. Both results support the significance of new extrahepatic lesion in PPS.

Currently, there is lack of effective second-line treatment for HCC patients with PD after sorafenib treatment. We showed that the main survival predictors of PPS include performance status, liver functions, early PD, and progression pattern. Understanding the survival predictors of PPS may have influence on clinical practice and guide physicians to refer patients for second-line trials or best supportive care. The subgroup analysis for PPS in candidates for second-line trials also identified tumor progression pattern as an important predictor and this should be taken into account by second-line trials design and patient recruitment.

This study has some limitations. First, it is a retrospective study. However, due to the strict reimbursement regulation of the National Health Insurance program in Taiwan, all patients received tumor reevaluation by contrast-enhanced CT or MRI every 2 months during sorafenib treatment. Second, the study enrolled only Asian population from Taiwan. Nevertheless, the impact of progression pattern on survival had only been reported in Western countries,^11^ and our results showed that the progression pattern determine the PPS not only in Western countries but also in Asian population. Third, regarding the reasons for sorafenib discontinuation, 34% of patients had concurrent PD and liver decompensation. It is our limitation to clearly define which one was predominant for them. Our data showed that not only liver decompensation alone but also concurrent PD and liver decompensation were worse predictor for OS and PPS. It looked that the clinical outcome of concurrent PD and liver decompensation was similar to liver decompensation alone.

In conclusion, the efficacy of sorafenib for advanced HCC in Taiwan was similar to the Asian-Pacific trial but was poorer than the results from Western countries. Performance status, liver functions, early disease progression, and progression pattern are important determinants of both OS and PPS. These factors should be considered in clinical practice as well as second-line trial designs for patients with PD after sorafenib treatment.
